# Hemoadsorption in Multiorgan Failure Due to Viscerocutaneous Loxoscelism

**DOI:** 10.3390/medicina61010143

**Published:** 2025-01-16

**Authors:** Raúl Valenzuela Córdova, David Rivera Estrella, José F. Bernardo, Darío Jiménez, Celia Rodríguez Tudero, Raúl Elías, José C. De La Flor

**Affiliations:** 1 Department of Nephrology, Hospital Nacional Cayetano Heredia, Lima 15002, Peru; carlos.valenzuela@upch.pe (R.V.C.); david.rivera@upch.pe (D.R.E.); jose.bernardo.c@upch.pe (J.F.B.); raul.elias.c@upch.pe (R.E.); 2 Faculty of Medicine, Peruana Cayetano Heredia University, Lima 15002, Peru; 3 Critical Nephrology Dialnef, Quito 170138, Ecuador; dario.jimenez@upch.pe; 4 Department of Nephrology, Hospital Universitario de Salamanca, 37007 Salamanca, Spain; crodrigueztudero@usal.es; 5 Surgery Doctoral Program, Faculty of Medicine, University of Salamanca, 37007 Salamanca, Spain; 6 Department of Nephrology, Hospital Central de la Defensa Gómez Ulla, 28047 Madrid, Spain; 7 Health Sciences Doctoral Program, Faculty of Medicine, Alcala University, 28805 Madrid, Spain; 8 Department of Medicine and Medical Specialties, Faculty of Medicine, Alcala University, 28805 Madrid, Spain

**Keywords:** loxoscelism, hemoadsorption, viscerocutaneous loxoscelism

## Abstract

*Background*: The bite of the Loxosceles spider is a public health problem around the world, mainly in Latin America. The viscerocutaneous presentation is related to the inoculation of toxins (phospholipase-D) that generates a systemic inflammatory reaction with a subsequent increase in cytokines and chemokines. Hemoadsorption is proposed as a therapy that allows for the removal of the toxin and modulation of the inflammatory response in this disease. *Case Report*: We present the case of a 31-year-old woman who was admitted to the hospital due to decreased urinary flow and jaundice 48 h after a spider bite. Despite treatment with intravenous (IV) monovalent antiloxoscelism serum, antibiotic therapy, and corticosteroids, the patient’s evolution was poor, and she was admitted to the critical care unit for severe multi-organ involvement, including hepatic and kidney damage and coagulation disorders, eventually requiring hemodialysis support and hemoadsorption therapy. After the therapy was administered, rapid improvement was evident with the suspension of vasopressor support and a decrease in inflammatory markers. *Conclusions*: This case presents hemoadsorption as a therapeutic option, based on its capacity to reduce the intensity of hyperinflammation and to regulate the immunological response.

## 1. Introduction

The bite of the Loxosceles spider is a public health problem around the world, mainly in Latin America. The spiders of the Loxosceles genus have a wide distribution with more than 140 known species; in South America there are three species: Loxosceles laeta (the most common in this region), Loxosceles intermedia (Brazil and Argentina), and Loxosceles gaucho (Brazil) [1]. The incidence of this event is around 200 cases per year in countries, such as Chile [2], but it can also go up to 559 cases per 100,000 habitants in some cities in Brazil [3]. The clinical manifestations are cutaneous loxoscelism (CL) and viscerocutaneous loxoscelism (VCL) [4]. CL is the most common presentation; it is reported in approximately 70% of the cases and it is characterized by edema, erythema, and skin necrosis in the area of the lesion. VCL is less common and is present in 1–27% of the cases, with systemic manifestations, such as intravascular hemolysis, disseminated intravascular coagulation, thrombocytopenia, liver compromise, and acute kidney injury (AKI) [5], which is present in up to 13% of cases and is the most common cause of death related to this condition [6].

The pathogenesis of the disease is related to the inoculated venom, which is made up of a complex molecular composition with peptides in the range of 5 to 45 kDa [7]. A group of toxins known as phospholipase-D (PLD) is common to all Loxosceles species. The key role of PLD was described for the first time 40 years ago when a hemolytic toxin that split sphingomyelin was isolated and named sphingomyelinase D, a denomination that has now changed to PLD, when it was proven that it also split other cellular phospholipids [8]. It also produces a local and systemic increase in cytokines and chemokines that can lead to complement activation with the migration of inflammatory cells and platelet aggregation. The presence of tumoral necrosis factor α (TNF-α) has been described in primary human keratinocytes cells [9], and the magnitude of the systemic response has been studied in animal models, in which the PLD administration reproduced a clinical presentation similar to septic shock with an increased release of TNF-α and interleukin-6 (IL-6); both elements would be involved in the manifestation of hypotension, metabolic acidosis, and neutrophil degranulation, but their specific role in the pathogenesis of VCL is unclear [10]. De Souza et al. reported, for the first time, the increase in IL-6 and TNF-α in cases of patients with loxoscelism [11].

The mechanism of CL is triggered by the action of PLD on the bite site. This molecule causes an increased inflammatory response that leads to tissular injury. Meanwhile, the VCL mechanism would be more complex and not clearly defined; PLD would play a main role in the systemic symptoms, while other toxins, like hyaluronidases and metalloproteinases, promote the spread of PLD to other tissues and systemic circulation, and they compromise distant organs [5], causing hematological alterations, such as intravascular hemolysis, thrombocytopenia, and disseminated intravascular coagulation (DIC). Another symptom is kidney failure, of which associated complications can lead to death. The exact behavior of this mechanism is not well established, but it is suspected that the development of acute tubular necrosis (ATN) caused by PLD could be responsible for it [12].

In relation to the treatment, there are no guidelines, and the current treatment options are controversial. The initial management includes wound cleaning with water and soap, the use of ice to inhibit PLD activity, and a tetanus vaccine. In the case of VCL, it is also necessary to control electrolytes and the hydric balance. The use of anti-loxosceles serum, systemic corticoids, dapsone, antibiotics, and surgical removal of the necrotic area around the lesion have been described as specific treatments [13]. It must be considered that even though anti-loxosceles serum is commonly used when available to inactivate the circulating venom [14], it has a variable efficacy depending on the spider species and the delay in administration, so it does not guarantee a good clinical course [15]. Even in Brazil where the serum is more widely used than in other countries, there are disagreements on the efficacy, and in experimental models, after 24 h of venom inoculation, the efficacy of the serum is reduced at 50% [14].

Therapeutic alternatives consider the use of therapeutic plasma exchange (TPE), mainly used in cases of refractory hemolytic anemia to remove cytokines, and immune complexes, which can theoretically remove the venom as discussed in case reports [16]. In one of these cases, a patient developed AKI and required hemodialysis support while also receiving TPE [16]. Another alternative that could remove cytokines is hemadsorption (HA), facilitating the control of the systemic inflammatory state that appears in VCL as observed in different acute inflammatory scenarios, such as in sepsis; however, the use of HA is controversial and cannot yet be recommended for patients with sepsis due to a lack of evidence [17,18]. HA enables blood purification due to its mechanism of adsorption based on mass separation using a solid agent or adsorbent. The adsorbent is produced as beads that have a large surface/volume ratio between a range of 300 to 1200 m^2^/g and the capacity to establish bonds, including ionic, hydrophobic, or Van der Waals bonds. Depending on the components of the sorbent, the beads may or may not be selective to some solutes to remove them from plasma once the blood has passed through the cartridge that contains the sorbent. An advantage of this modality of blood purification is that could be used simultaneously with a wide range of techniques of renal replacement, such as hemodialysis (HD), sustain low-efficiency dialysis (SLED), or the continuous renal replacement therapies (CRRT) that is needed [19].

We present the case of a patient with VCL who developed severe multi-organ involvement, including hepatic and kidney damage and coagulation disorders. The patient was treated with intravenous (IV) monovalent anti-loxosceles serum, antibiotic therapy, corticosteroids, vasoactive drugs, and HA associated with SLED. The patient had a favorable clinical evolution. This case considers the possible benefits that HA can provide in the classic management approach of these patients.

## 2. Case Report

**Figure 1:**
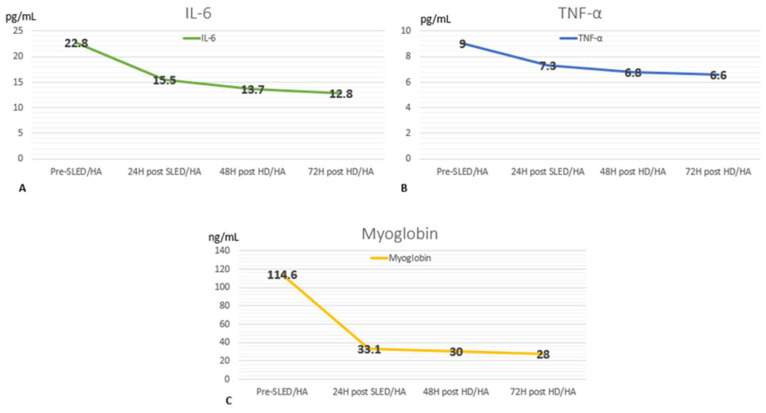
Changes in serum levels of IL-6 (interleukin 6) (**A**), TNF-α (tumor necrosis factor-α) (**B**), and myoglobin (**C**) during pre and post (24, 48, and 72 h (hours)) sessions of SLED (sustained low-efficiency dialysis) or HD (hemodialysis) associated with HA (hemoadsorption).

We present the case of a 31-year-old Latina woman, an agricultural worker with no relevant medical history. She initially sought care at a primary health center after suffering a spider bite on her left dorsolateral region 48 h earlier. Her condition deteriorated and she presented generalized jaundice and oliguria. Initial laboratory blood tests revealed urea levels of 125 mg/dL, serum creatinine (sCr) of 1.82 mg/dL, total bilirubin (TB) of 21.6 mg/dL with indirect bilirubin (IB) predominance of 16.84 mg/dL, a urine test of 8–10 red blood cells, and the detection of hemoglobin +3 (all other laboratory findings are shown in Table 1). Due to the severity of her condition, she was referred to our hospital and admitted to the emergency department. On admission, her clinical condition was assessed as fair. The physical examination revealed a body mass index (BMI) of 25.3 kg/m^2^, a heart rate of 63 beats per minute, a respiratory rate of 17 breaths/min, blood pressure of 95/60 mmHg, fever of 38 °C, and an ambient oxygen saturation of 95%. The skin showed generalized jaundice, and on the left dorsolateral region, there was a painful violaceous plaque of 6 × 4 cm with necrosis in the central region. The rest of the evaluation was normal, and the complementary test showed hemoglobin at 11.9 g/dL, white blood cells (WBCs) at 15,920 mm^3^, urea at 232 mg/dL, SCr at 5.51 mg/dL, TB at 19.4 with IB predominance of 17.4 mg/dL, arterial blood gas, metabolic acidosis, and respiratory alkalosis; a new urinary test showed 100 leucocytes, 2–4 red blood cells, hemoglobin +3, and bilirubin +1 (Table 1). The patient was evaluated by the service of Tropical and Infectious Diseases, which established the diagnosis of viscerocutaneous loxoscelism, and she received treatment that included hydration with saline serum at 150 mL/h, IV monovalent anti-loxosceles serum, IV clindamycin 1.8 g/day divided into four equal doses, IV ceftriaxone 2 g IV once/day, and IV dexamethasone 4 mg IV twice/day. Despite this, the clinical evolution was unfavorable after 12 h of admission, with a diuresis of 0.3 mL/kg/h and oxygen requirement by binasal cannula at 4 liters (L) per minute, plus hemodynamic instability. She was transferred to the Intensive Care Unit (ICU) due to multiorgan dysfunction with kidney, liver, endothelial, and hematological compromise, with an initial Acute Physiology, Age and Chronic Health Evaluation (APACHE) II score of 13 and Sequential Organ Failure Assessment (SOFA) score of 9. In the ICU, she started receiving vasopressor support with norepinephrine 0.12 ug/kg/h and hydrocortisone 100 mg/8 h. The renal function continued to deteriorate with an increase in urea and creatinine and the presence of metabolic acidosis and hemodynamic instability. Therefore, the clinical condition of AKI stage 3 Kidney Disease Improving Global Outcomes (KDIGO) 2012 led to the initiation of sustained low efficiency dialysis (SLED) with the following prescription: periods of 6 h with a dialysis dose calculated via the Daugirdas modified equation for an initial reduction of urea by 35%. The filter was Nipro Elisio Polynephron™ (NIPRO CORPORATION, Osaka, Japan) 1.7 m^2^; simultaneously, HA of 4 h was initiated with a Jafron HA330© cartridge (JAFRON BIOMEDICAL CO., LTD., Zhuhai, China). Before starting therapy, the patient and family members were informed about the therapy to be performed, its risks, and the benefits, after which the informed consent was signed. Serum levels of TNF-a, myoglobin, and IL-6 were measured prior to the start of treatment (Figure 1). This procedure was repeated after the end of HA. There were no complications during the extracorporeal treatment, and the oxygen requirement was reduced, the vasopressors were suspended 3 h after SLED-HA was over. The inflammatory biomarkers measured in blood were reduced. The patient remained in the ICU for two more days and received two sessions of HA with a Jafron HA330© cartridge for 3 h, plus 4 h of conventional hemodialysis (HD) for 2 consecutive days; the inflammatory biomarkers were monitored at 24, 48, and 72 h (Figure 1), with a subsequent APACHE score of 7. The patient received six inter-day conventional HD sessions due to persistent kidney injury, with a posterior improvement of diuresis from 0.4 mL/kg/h to 1 mL/kg/h and an increase in renal function. The skin lesion had mild extension progression with the development of a scab after 10 days. The patient was discharged 18 days after being bitten by the spider with a favorable prognosis.

## 3. Discussion

We present the first ever reported case of VCL with severe multi-organ involvement, including hepatic and kidney damage and coagulation disorders, treated with HA associated with HD and support therapy with a good clinical and analytical response to the prescribed treatment. Our patient presented VCL with an initial presentation of a skin lesion that was a painful purplish plaque with necrosis in the central region, progressive jaundice, fever, and reduced urinary outflow 48 h after the bite. Maher et al. reported a series of cases of VCL with systemic involvement, where the onset of symptoms ranged from 1 to 10 days after the bite [14]. The presence of jaundice and fever is highly suggestive of hemolytic anemia, and in some retrospective studies, the mean time until presentation was 2 days, very similar to our case, although the manifestations of hemolytic anemia could appear up to 7 days after the bite. The reduced urinary flow indicates the compromise of the kidney function [5]. In a cross-sectional study in Brazil, all patients with AKI had fever and skin lesions with erythema, but only 30% had jaundice [6]. This is different from our case due to the appearance of marked jaundice. Hypotension is also an uncommon manifestation but is the principal form of cardiac disorder according to the cases reported by Gremski et al. [5].

Regarding blood test analytics, our case presented clear signs of hemolytic anemia, with the presence of decreased hematocrit from 42% to 27% and elevated total bilirubin and LDH. These findings are to be expected according to Gremski’s review, which also describes hemolytic anemia with the presence of proteinuria, hemoglobinuria, and bilirubinuria [5], very similar to our case. Our patient had AKI stage 3 according to the KDIGO 2012 classification [20] with increased creatinine, urea, and metabolic acidosis. Patients with AKI are more commonly associated with rhabdomyolysis and hematuria, but in our patient, the urinary alterations were proteinuria and pyuria, which are rare in stage-3 AKI due to Loxoscelism [5]. Compromise of the liver was subsequently seen with an increase in aspartate aminotransferase (AST) and alanine aminotransferase (ALT). The alteration in the prothrombin time (PT) and thromboplastin time (PTTa) could be sign of the dysregulation of coagulation caused by the toxins of the venom. In our case, the patient had, besides these findings, increased levels of direct bilirubin, which revealed compromise of the liver [5].

The established treatment was the administration of IV fluid therapy, systemic corticoids, antibiotics, and anti-loxosceles serum. Despite this, the clinical evolution of our patient was poor, perhaps due to the fact that the possible causal agent was Loxosceles laeta (higher rate of cutaneous–visceral involvement associated with the venom of this spider), in addition to the fact that the bite was located on the trunk (highly vascularized region) and finally the delay in medical attention and consequently the application of anti-loxoceles serum [21,22], needing support with norepinephrine, oxygen, and kidney replacement therapy. The recommended kidney replacement therapy in a hemodynamically unstable patient is usually CRRT [23], although the use of SLED is also an accepted modality in critically ill patients [24]. In order to control the hyper-inflammatory state produced by the loxosceles bite, it was decided to couple HA to SLED therapy, considering the similarity of an inflammatory state to that of septic shock [10], and the experience of this hybrid therapy coupled with HA in our center is favorable in critical patients with sepsis caused by pancreatitis and leptospirosis with a reduction in inflammatory biomarkers and without technical complications [25], so we indicated a session of HA associated with SLED until hemodynamic stability was achieved, the hyper-inflammatory state was controlled, and inflammatory biomarkers were reduced. Subsequent HD with two more HA sessions was indicated to keep IL-6, TNF-α, and myoglobin levels under control and to minimize the potential rebound effect due to the persistence of the inflammatory cascade that was established with the initial bite injury. We used the Jafron HA cartridge, first because of its availability in our unit and second because of its potential benefit of the increased cytokine killing capacity. We did not consider selective cartridges for sepsis because they are targeted for the elimination of endotoxins [15], derived from some bacterial agent [26]. Other selective cartridges are used in autoimmune diseases, so we opted for a non-selective HA, having Cytosorb^®^ and HA Jaffron cartridges available. Several studies have evaluated the results of each type of cartridge in different types of renal replacement therapy and diseases with systemic inflammation. For example, the different modalities of CRRT had been paired with HA in studies reviewed by Ricci et al. [27] to control a dysregulated immune response mainly due to sepsis. It has been seen in prospective studies that patients with refractory septic shock with values of IL-6 above 1000 ng/mL that were treated with CRRT and non-selective HA with a Cytosorb^®^ cartridge and patients with COVID-19 infection with vasoplegic shock treated with the same extracorporeal therapy combination showed inconclusive results regarding the IL-6 reduction, perhaps due to the stratification of the study population [27]. Despite these results, other studies that evaluated HA in patients with respiratory insufficiency and a COVID-19 infection showed a favorable clinical outcome in the group of HA regarding the oxygen level and reduction in cytokines [28], with a favorable trend with the use of Jafron 330 HA versus Cytosorb^®^ [29]. The use of the Jafron 330 HA cartridge has also been reviewed in case series presented by Sazonov et al. [30], with three pediatric patients with oncological disease that developed sepsis and AKI, which were managed in the pediatric ICU with continuous venovenous hemodiafiltration (CVVHDF) and HA. Although the CVVHDF Primaflex filter can partially eliminate IL-6, IL-10, and TNF-α, the use of at least one HA session was used for a more effective reduction in inflammatory biomarkers. A more efficient reduction in IL-6 was also described with the Jafron 330HA cartridge than with Cytosorb^®^. Two of the patients of the case series had a favorable clinical evolution and were discharged from the pediatric ICU, and one of them died due to oncological complications [30]. Other studies also support the apparent benefits of the Jafron HA330 in the reduction of the cytokine load in the plasma and lung tissue with a reduced length of stay at the ICU and mortality [31]. Despite the evidence presented on the use of HA in patients with systemic inflammation, there is a low level of evidence. A recent meta-analysis including six articles evaluating the use of Cytosorb^®^ in patients with sepsis found no difference in mortality at 28–30 days. In secondary outcomes, such as the use of vasopressors or a reduction in inflammatory markers, there is evidence of great variability in clear benefits, concluding that they do not recommend the use of HA outside clinical studies [18].

The IL-6 reduction could be a key element in the physiopathology of VCL, and this could also account for the favorable evolution of our patient after this reduction. Because of the apparent greater reduction in IL-6, as well as the clinical results mentioned previously and our center experience, it was decided to use the cartridge Jafron HA330© that has the capacity to absorb middle-sized molecules between 6 to 26 kDa [32], which explains the reduction in IL-6, myoglobin, and TNF-α by 32%, 71%, and 19%, respectively. In the case of TNF-α, this molecule is 17 kDa as a soluble monomer or 51 kDa as a homotrimer attached to the cell membrane, which could affect the absorption capacity of the cartridge, as also seen in the case published by Onuk et al. [32,33]. The reduction in TNF-α and IL-6 would theoretically reflect a decrease in the inflammatory state.

There is no prior evidence of the use of HA as therapy in the management of Loxoscelism so there is no reference regarding the HA prescription as in sepsis cases, although theoretically, PLD and other toxins, such as metalloproteinases and hyaluronidases that are present in the Loxosceles venom, could be absorbed [34], which help to control the inflammation cascade and then continue to flow through the blood circuit to the HD filter. In this case, we extrapolated the duration and specifications according to the patient’s clinical evolution and studies of critically ill patients due to septic shock or a COVID-19 infection, with a final period of 3 days [19]. Among the limitations of HA, it should be considered that it could remove the antimicrobial agents necessary for treatment, as well as biocompatibility problems that could generate the consumption of hematological elements [19], mainly platelets [35], although this is less frequent at present. It should be considered that the non-selective elimination of cytokines could have an unbalancing effect on immunity, so the tolerance of the session should always be assessed [35]. Other favorable elements, such as anti-inflammatory mediators or plasma amino acids could also be removed, although the main element removed would be toxic [19]. A positive outcome after the therapy is uncertain due to the characteristics of a critically ill patient as seen in reviews of this therapy with some studies on favorable and unfavorable mortality outcomes [19] The requirement of at least three HA sessions to obtain results should also be considered in the cost of this therapy.

Other modalities of extracorporeal therapy will be the TPE performed to manage hemolytic anemia cases of patients bitten by a brown recluse spider, in patients with poor responses to corticoids, and, in one case, with a poor response to intravenous immunoglobulin (IVIG); in both cases, they kidney function was mildly compromised or preserved, and no renal replacement therapy was required; after TPE was conducted, the hemoglobin levels stabilized at both patients were discharged [15,36]. We considered that prescribing HD and then TPE required albumin or plasma, which therefore could increase the risk of losing clotting factors and being less efficient than a simultaneous therapy achieved with SLED and HA [19].

The favorable clinical response after HA and the serum values of inflammatory markers, which were not excessively high, allowed us to consider an intervention at the right time to help the patient recover. The HA performed alone or simultaneously with other extracorporeal therapies, such as intermittent HD, SLED, and other CRRT provides benefits in the management of critically ill patients, with a key role in hemodynamic stabilization, proinflammatory cytokine clearance, and regulation of the immune response [25]. However, the limitations of this therapy should always be considered before it is initiated, such as the theoretical control of the inflammatory state by reducing TNF-α and IL-6 levels. Despite the advantages of HA seen in this case, the use of this extracorporeal therapy should be performed with caution considering that HA was not previously designed or used in patients with VCL. The lack of studies that ensure similar results in this type of patient requires further research, with studies of a higher level of evidence to really prove the efficacy of this therapy in cases of VCL.

## 4. Conclusions

This case considers HA as a therapeutic option, based on its capacity to reduce the intensity of hyperinflammation and to regulate the immunological response. It should also be considered that HA could help to overcome the limitations of the current management alternatives. The HA shares with TPE the principle that it can “clear” molecules that contribute to maintaining an inflammatory state. However, only HA can be performed simultaneously with HD depending on the patient’s needs, and it does not require exchange with other solutions, like plasma or albumin. This could be crucial to setting a more favorable prognosis for the patients. However, studies with a higher level of evidence are needed to prove the efficacy of this therapy in these VCL patients.

## Figures and Tables

**Table 1:** Laboratory findings.

	Day 1	Day 2	Day 3	Day 6	Day 10	Day 18	Normal Values
Hematocrit (%)	42	35	32	28	26	27	36–44
White blood count (/mm^3^)	10,700	15,290	10,990	10,390	8090	6920	4000–12,000
Platelets (10^3^/mm^3^)	180	173	160	196	258	321	150–400
Serum glucose (mg/dL)	134	173	178	120	76	80	70–125
Serum urea (mg/dL)	125	232	150	97	63	62	5–20
Serum creatinine (mg/dL)	1.82	5.51	5.08	4.45	2.35	1.5	0.6–1.1
Serum sodium (mEq/L)	132	135	136	137	138	141	135–145
Serum potassium (mEq/L)	4.8	4.38	4.9	4.5	4.3	4.38	3.5–5.5
Serum chloride (mEq/L)	100	101	101	100	97	104	96–106
Serum Ionic calcium (mmol/L)	-	0.95	1.05	1.2	1.18	1.1	1.15–1.29
Alanine transaminase (U/L)	158	29	44	32	24	22	4–36
Aspartate transferase(U/L)	195	32	45	41	38	24	8–33
Alkaline phosphatase (U/L)	80	64	59	72	130	117	44–147
Prothrombin time (seconds)	-	30	14.7	14.3	13.7	16,5	11–13.5
PTTa (seconds)	-	38	37.4	29	30	34	25–35
INR	-	2.93	1.05	1.02	1	1.1	0.8–1.1
Total bilirubin (mg/dL)	21.61	19.4	8.9	5.1	1.3	1	0.1–1.2
Indirect bilirubin (mg/dL)	16.81	17.4	7.5	4	1.1	0.5	0.2–0.8
Serum Albumin (g/dL)	-	2.8	2.4	2.5	-	2.9	3.4–5.4
LDH (UI/L)	-	2151	1593	523	320	290	140–280
Ph	-	7.25	7.3	7.46	-	7.48	7.37–7.42
HCO_3_ (mmol/L)	-	20.3	17	22	-	27	22–29
pCO_2_ (mmHg)	-	14	27	29	-	36	35–45
pO_2_ (mmHg)	-	115	100	89	-	90	75–100
Oxygen saturation (%)	-	93	95	94	-	97	95–100
Fraction inspired O_2_ (%).	-	21	30	21	-	21	21
Lactate (mmol/L)	-	0.6	0.8	-	-	1.2	<1.6
Urine pH	-	4	6	-	-	5	4–7
Urine density	-	1025	1015	-	-	1015	1005–1030
Urine hemoglobin	-	+3	+3	-	-	Neg.	Neg.
Urine bilirubin	-	Neg.	+2	-	-	Neg.	Neg.
Albuminuria	-	+2	+3	-	-	Neg.	Neg.
Urine leucocytes per HPF	-	6–10	>100	-	-	2–4	<5
Urine red blood cells per HPF	-	8–10	2–4	-	-	1–2	<2

PTTa (partial thromboplastin time), INR (international normalized ratio), LDH (lactate dehydrogenase), HCO_3_ (bicarbonate), pCO_2_ (partial pressure of CO_2_), pO_2_ (partial pressure of O_2_), HPF (high-power field), Neg.: negative.

## Data Availability

No new data were created or analyzed in this study. The data used to support the findings of this study are available from the corresponding author on request (contact, J.C.D.L.F., jflomer@mde.es).
